# Supplementary material for: Unraveling distinct effects between CuOx and PtCu alloy sites in Pt−Cu bimetallic catalysts for CO oxidation at different temperatures
Source: Nat Commun. 2024 Jul 3;15:5598. doi: 10.1038/s41467-024-49968-6 (PMC11222431; doi:10.1038/s41467-024-49968-6)
Supplement: Supplementary file 1 — Supplementary Information [file 41467_2024_49968_MOESM1_ESM.pdf]

Supporting Information

Unraveling Distinct Effects between CuO_x and PtCu alloy Sites in Pt−Cu Bimetallic Catalysts for CO Oxidation at Different Temperatures

Yunan Li[1,2,3 ‡], Lingling Guo[2 ‡], Meng Du[1,2], Chen Tian[1,3], Gui Zhao[4], Zhengwu Liu[1,3], Zhenye Liang[1,3], Kunming Hou[2], Junxiang Chen[5], Xi Liu[4], Luozhen Jiang[1,2]*, Bing Nan[1,2]*, Lina Li[1,2]*

[1] *Shanghai Institute of Applied Physics, Chinese Academy of Sciences, Shanghai 201204, China*

[2] *Shanghai Synchrotron Radiation Facility, Zhangjiang Laboratory, Shanghai Advanced Research Institute, Chinese Academy of Sciences, Shanghai 201210, China*

[3] *University of Chinese Academy of Sciences, Beijing 100049, China*

[4] *School of Chemistry and Chemical, In-situ Centre for Physical Sciences, Shanghai Jiao Tong University, Shanghai 200240, China*

[5] *Division of China, TILON Group Technology Limited, Shanghai 200090, China*

*Corresponding author. E-mail: nanb@sari.ac.cn (B. Nan); jianglz@sari.ac.cn (L. Jiang); lilina@sinap.ac.cn (L. Li).

‡ These authors contributed equally to this work.

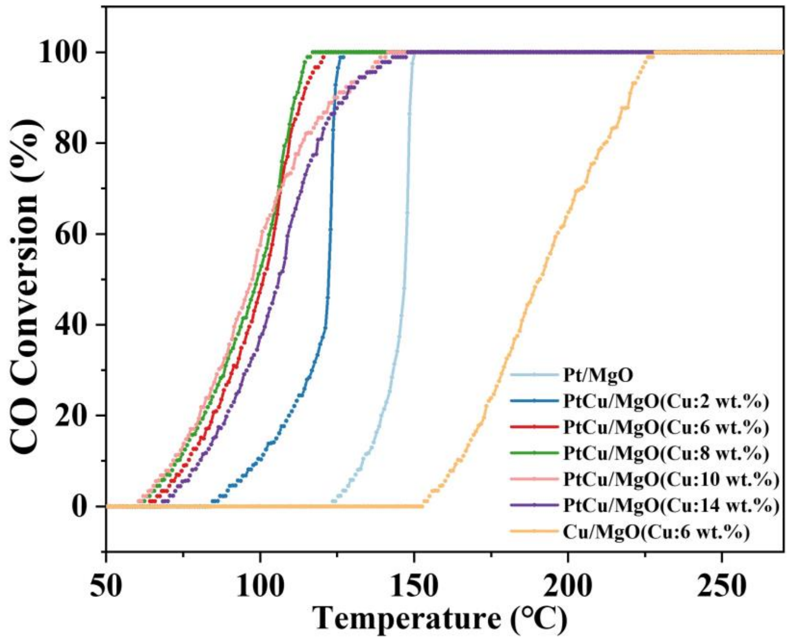

Supplementary Fig. S1. "Light off" profiles for CO Oxidation reaction (1%CO/20%O_2/79%He, 120,000ml·h^{-1}·g_{cat}^{-1}).

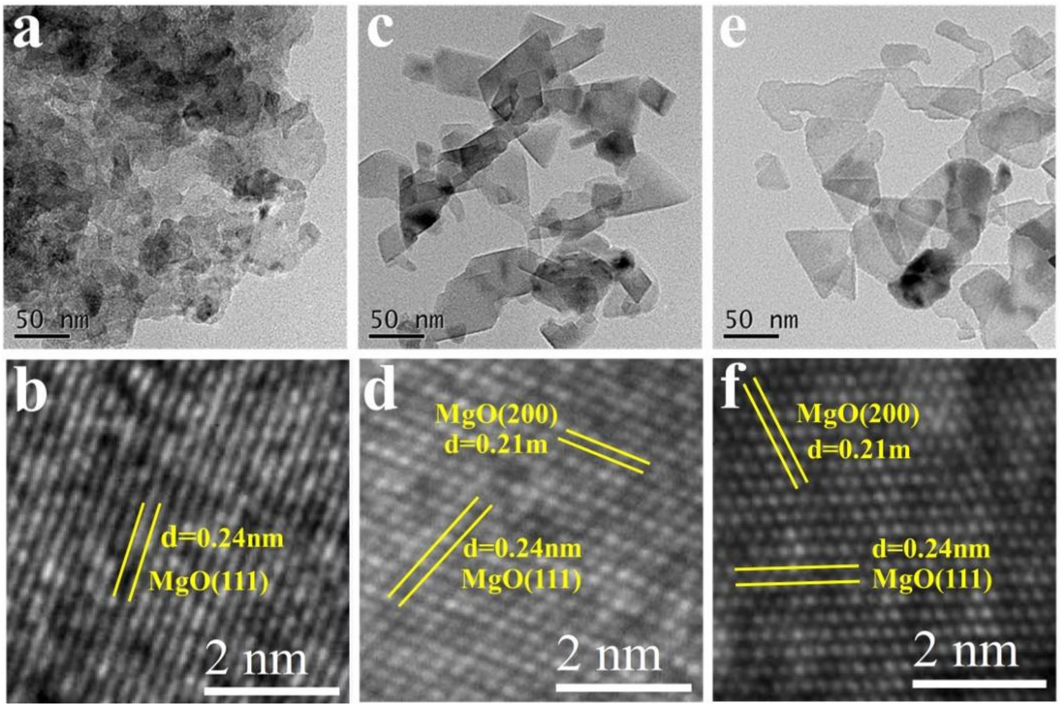

Supplementary Fig. S2. TEM and HRTTM images of catalysts: (a, b) Pt/MgO, (c, d) Cu/MgO, (e, f) PtCu/MgO.

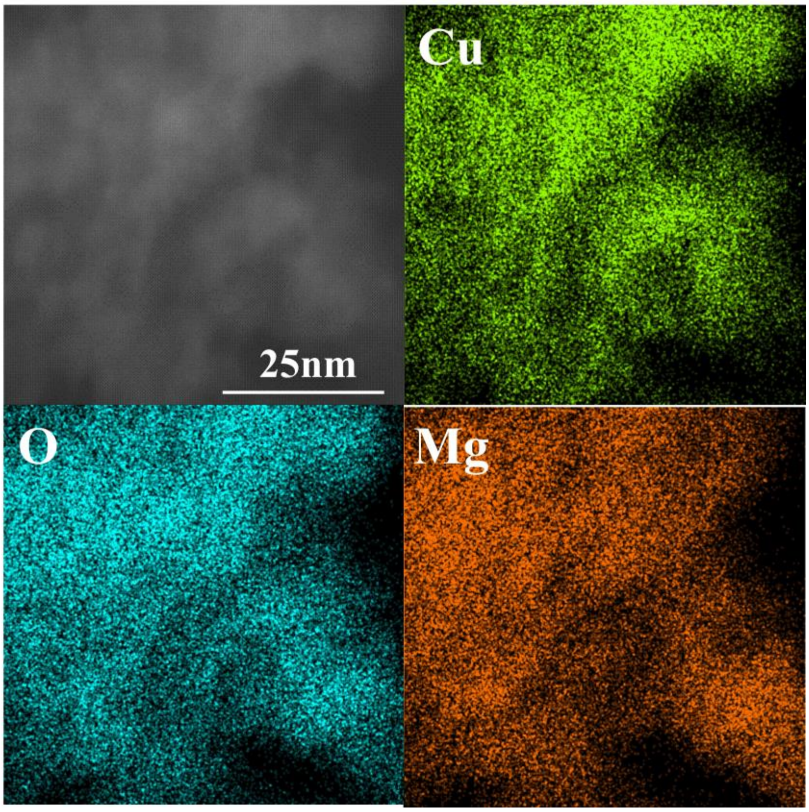

Supplementary Fig. S3. EDS mapping results of Cu/MgO catalyst.

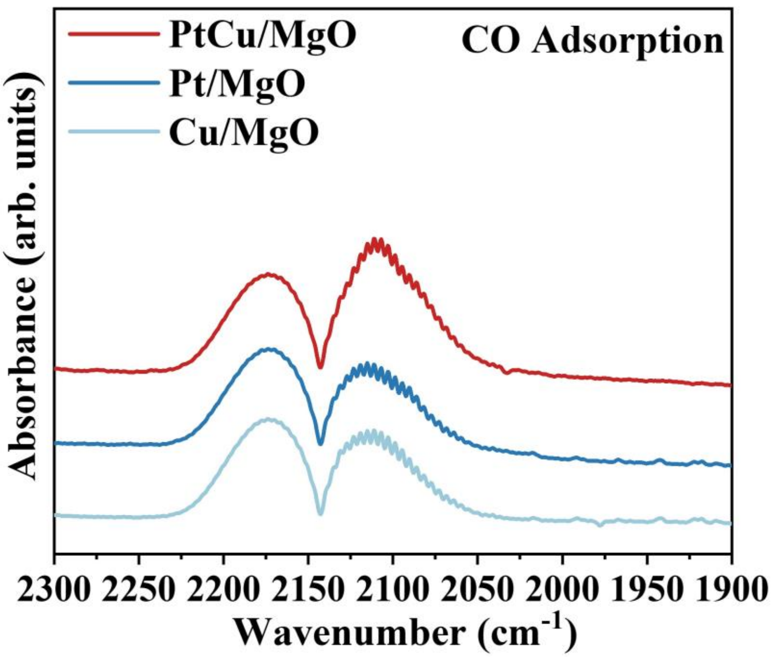

Supplementary Fig. S4. In-situ DRIFTS spectra for PtCu/MgO catalysts tested at 100°C after oxygen pretreatment: The catalysts were pretreated in situ at 300 °C under O_2 flow in the DRIFTS reaction cell before data collection.

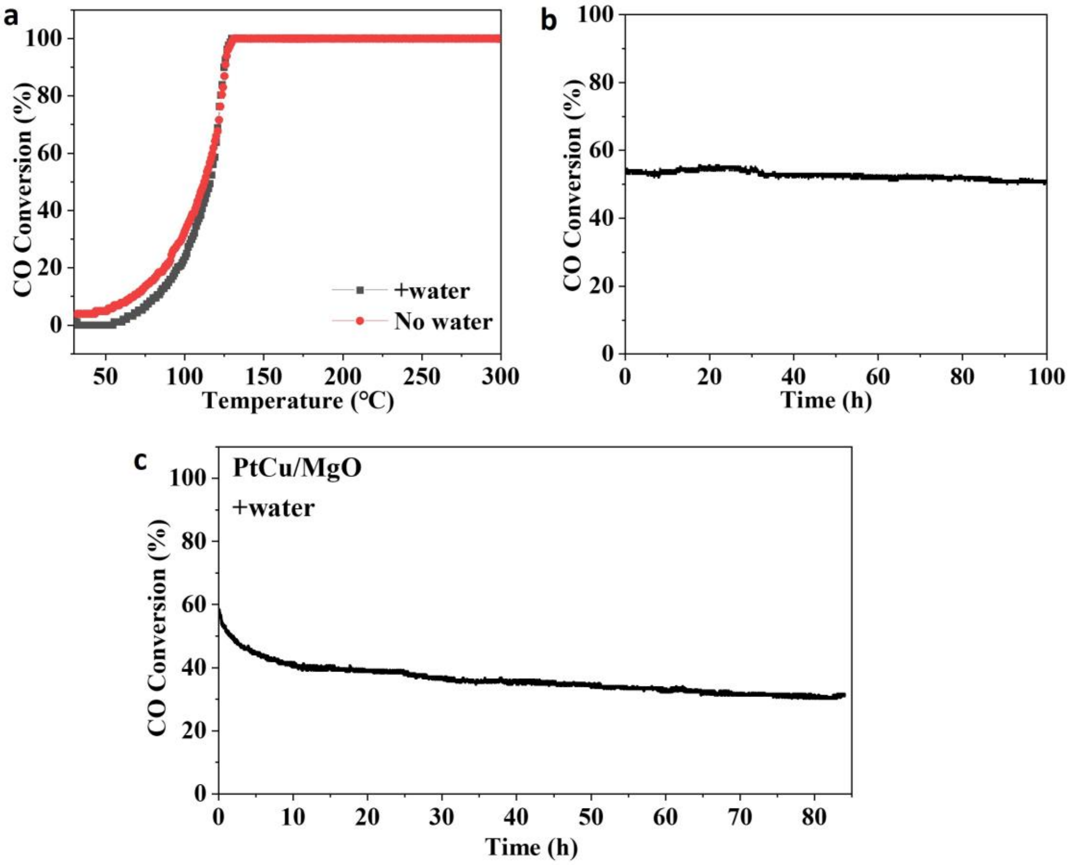

Supplementary Fig. S5. (a) Catalytic performance for PtCu/MgO under the presence and absence of water vapor condition (1vol.%CO/20%O_2/79%He, 120,000 ml·h^{-1}·g_{cat}^{-1}), (b) Stability test of PtCu/MgO at 115°C for CO oxidation reaction, (c)Water vapor stability experiments for PtCu/MgO catalysts at 120°C.

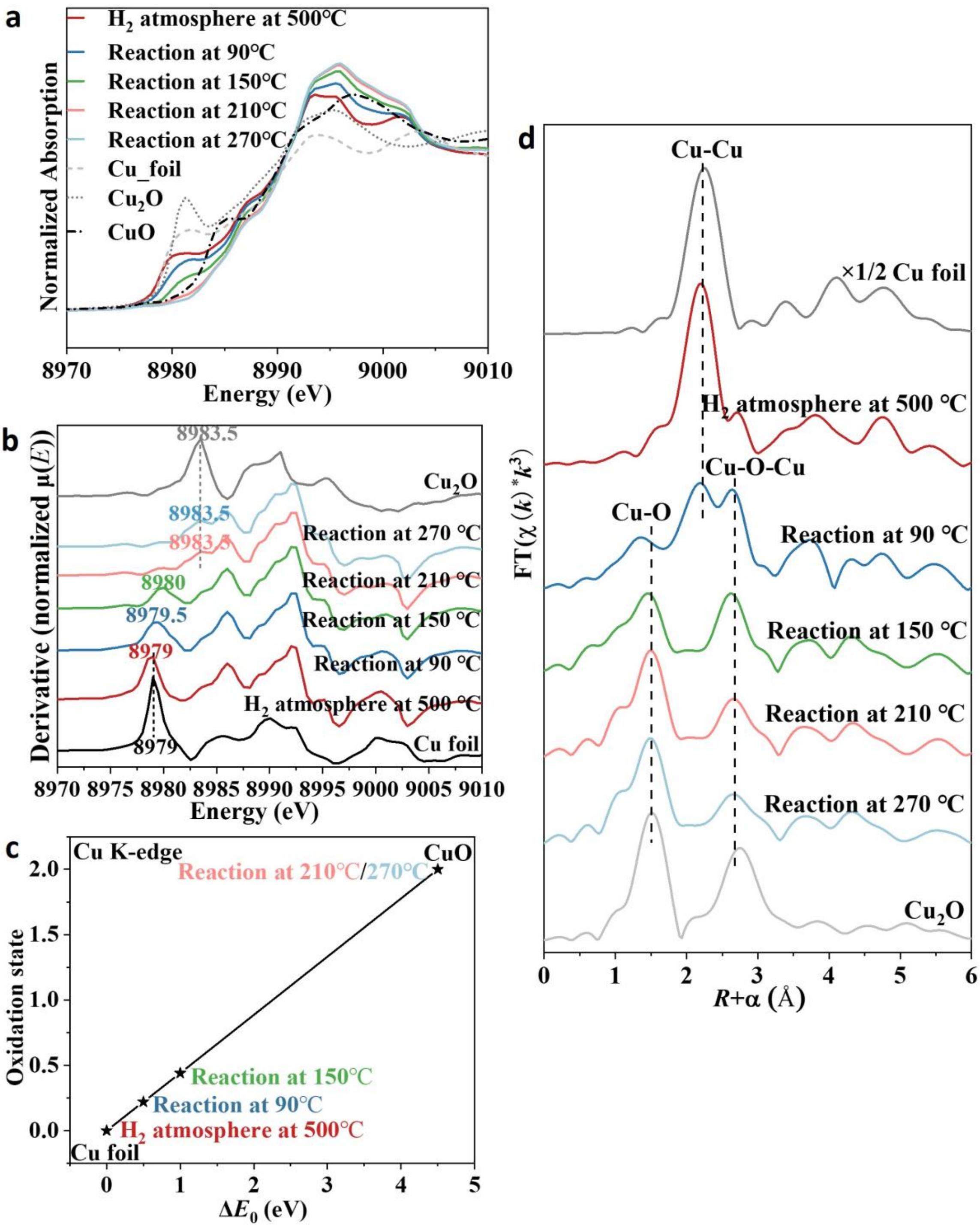

Supplementary Fig. S6. (a) in-situ Cu K-edge XANES profiles, (b) The Cu K-edge Derivative XANES spectra of PtCu/MgO catalysts and the references, (c) the average oxidation state of Cu in PtCu/MgO catalysts from XANES spectra, (d) in-situ Cu K-edge EXAFS profiles (the data are k^3-weighted and not phase-corrected).

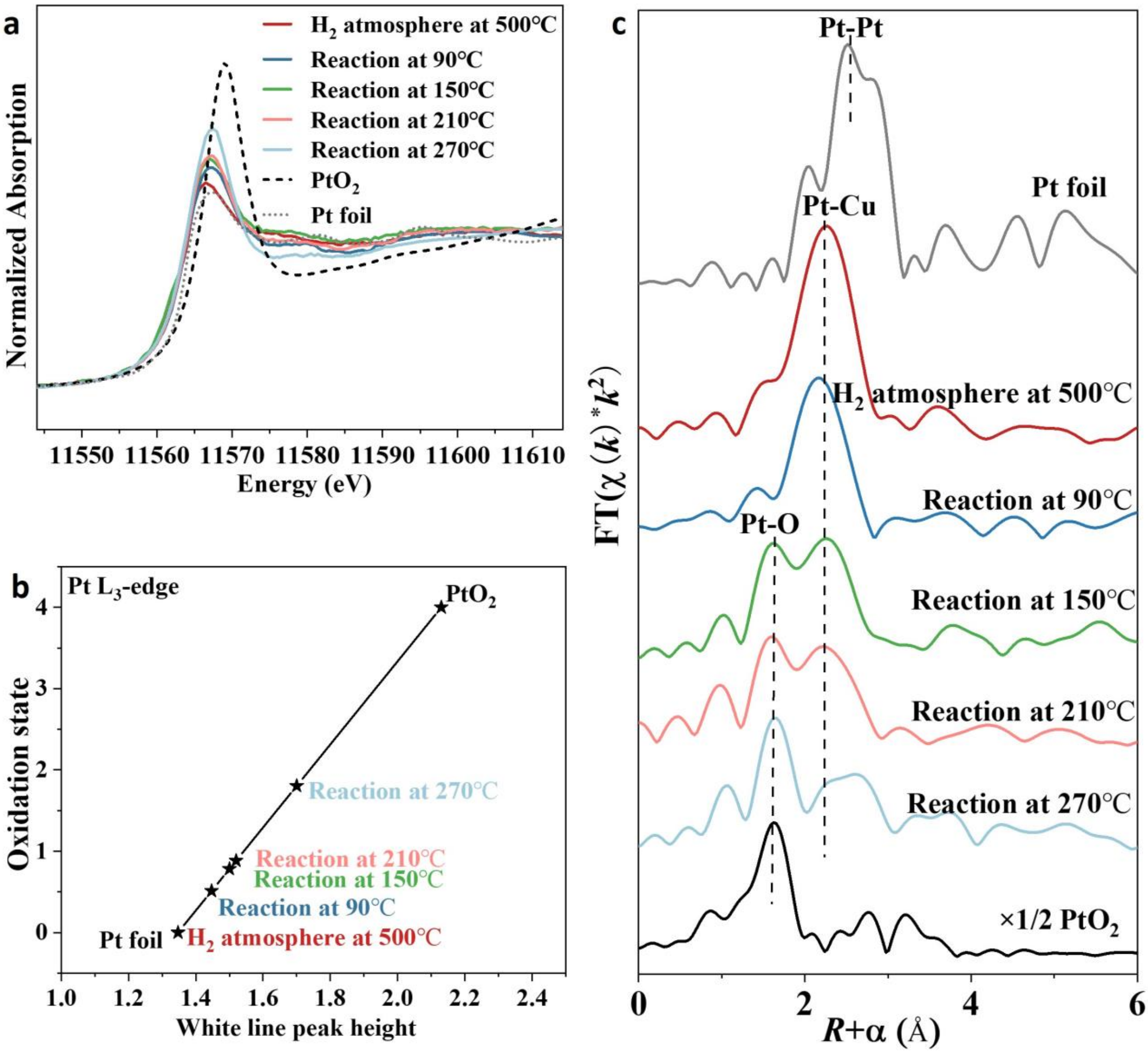

Supplementary Fig. S7. (a) in-situ Pt L_3-edge XANES profiles, (b) the average oxidation state of Pt in PtCu/MgO catalysts from XANES spectra, (c) in-situ Pt L_3-edge EXAFS profiles (the data are k^2-weighted and not phase-corrected).

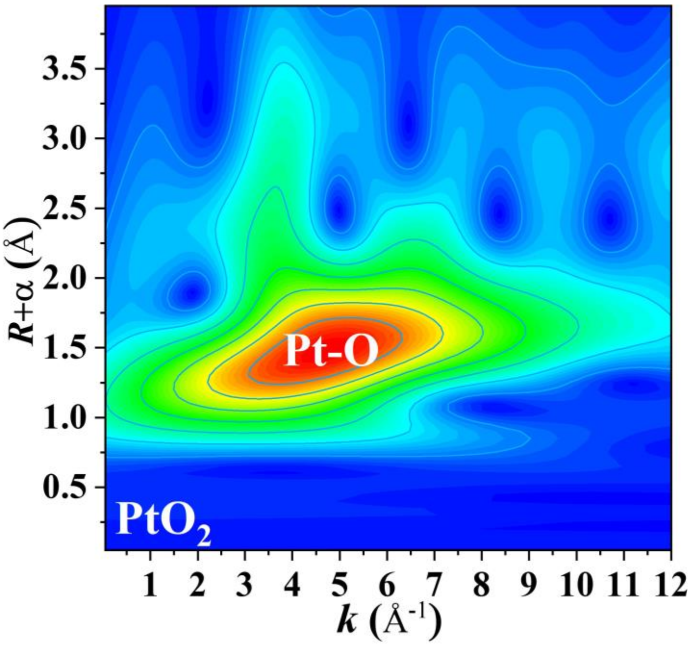

Supplementary Fig. S8. WT-EXAFS contour plot of Pt L_3-edge signals for PtO_2.

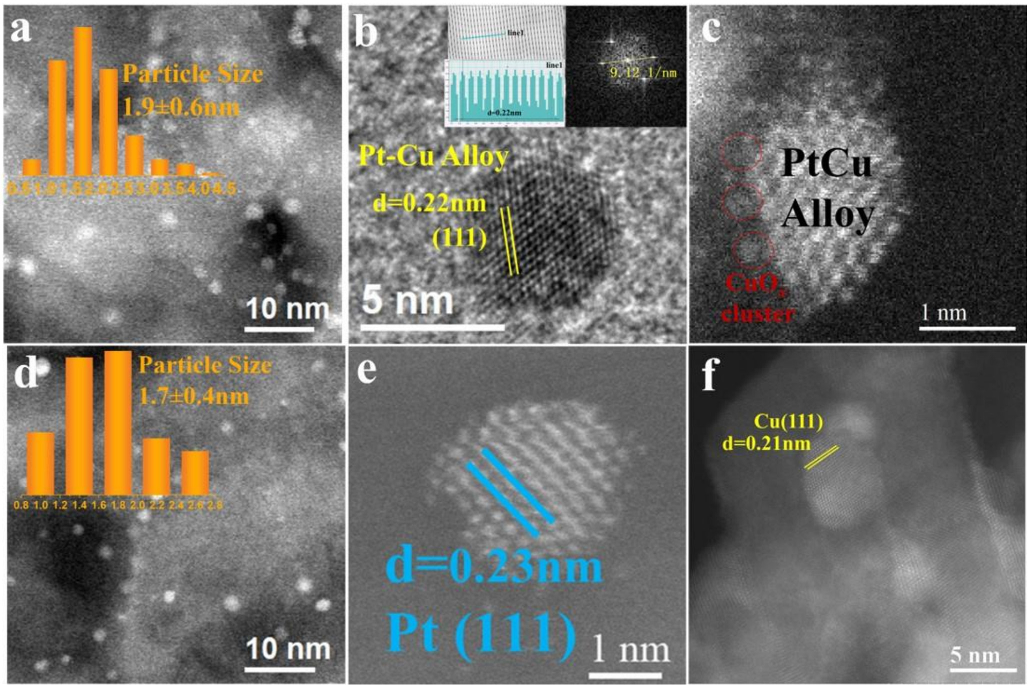

Supplementary Fig. S9. Representative aberration-corrected HAADF-STEM images and EDS mapping results of PtCu/MgO catalysts: (a-c) PtCu/MgO (used); (d-e) Pt/MgO (used); (f)Cu/MgO (used).

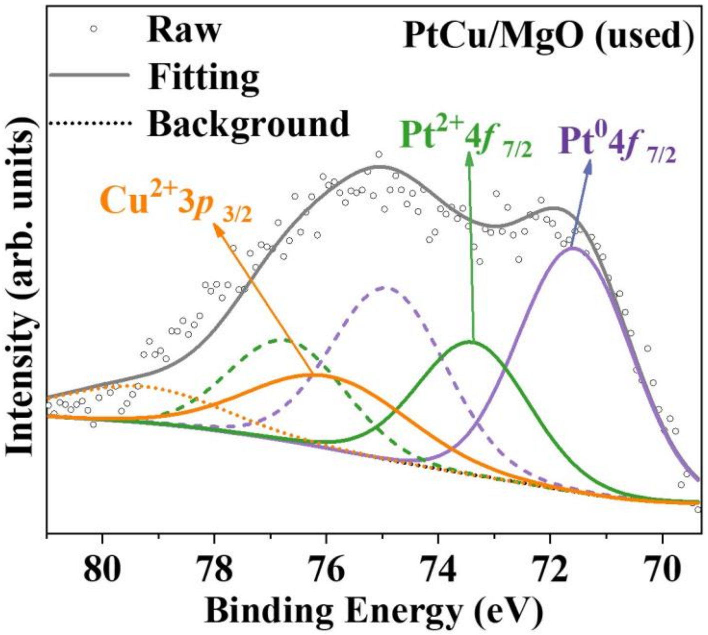

Supplementary Fig. S10. XPS spectra of Pt 4*f* for PtCu/MgO after catalytic CO oxidation.

The Cu 3*p* contribution should be taken into account when fitting Pt 4*f* spectra due to the overlapping of the peaks of Cu 3*p* and Pt 4*f*. The XPS spectrum for PtCu/MgO(used) was fitted using three doublets: first at 71.5 eV corresponding to Pt^0, second at 73.4 eV corresponding to Pt^{2+} and third at 76.1 eV corresponding to Cu^{2+}.

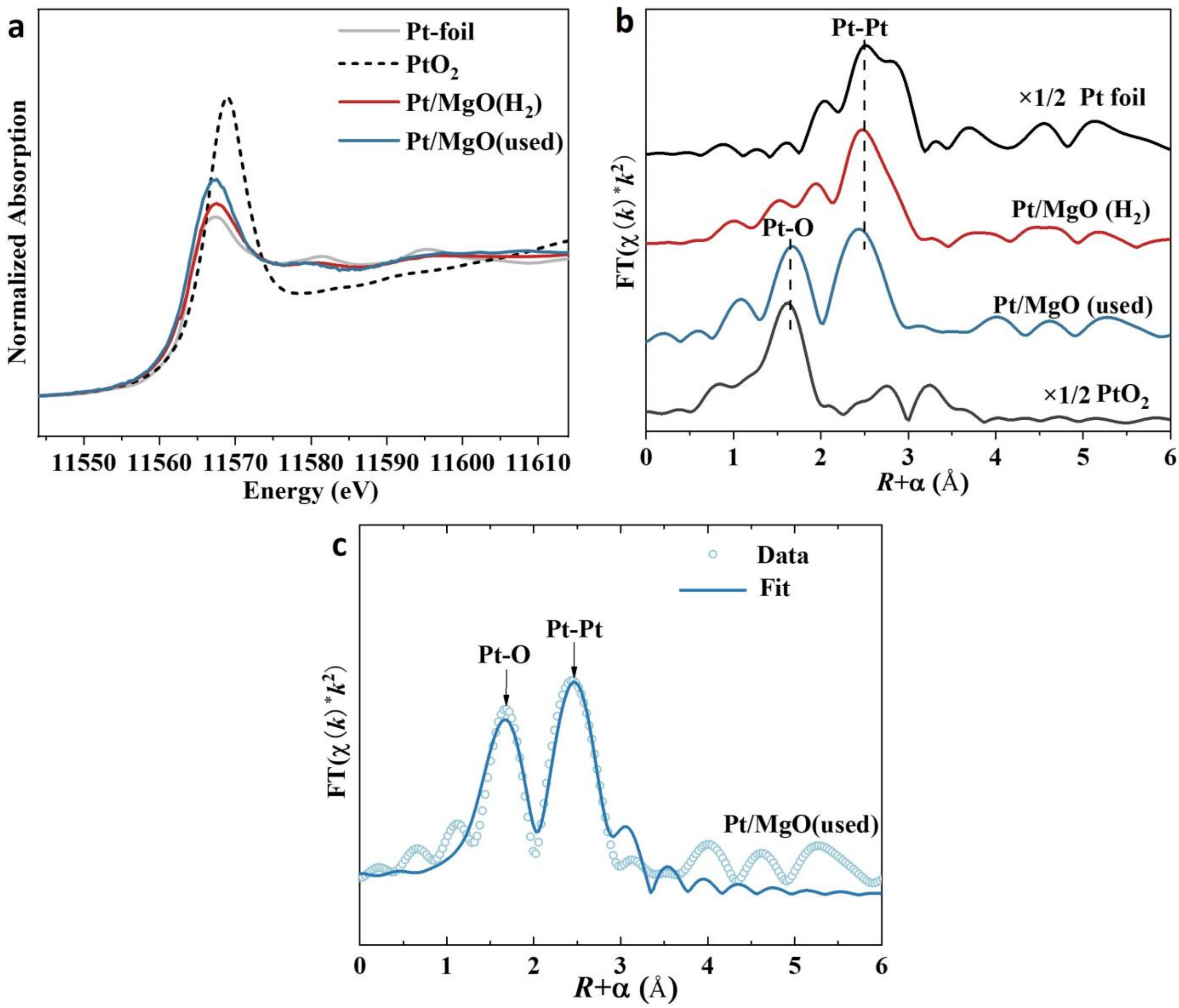

Supplementary Fig. S11. (a) Pt L_3-edge XANES profiles, (b) Pt L_3-edge EXAFS profiles of Pt/MgO catalyst, (c) Pt L_3-edge XAFS (points) and cure-fit (line) of PtCu/MgO (used). (The data are k^2-weighted and not phase-corrected, the curve-fit was generated using the parameters in Supplementary Table S2).

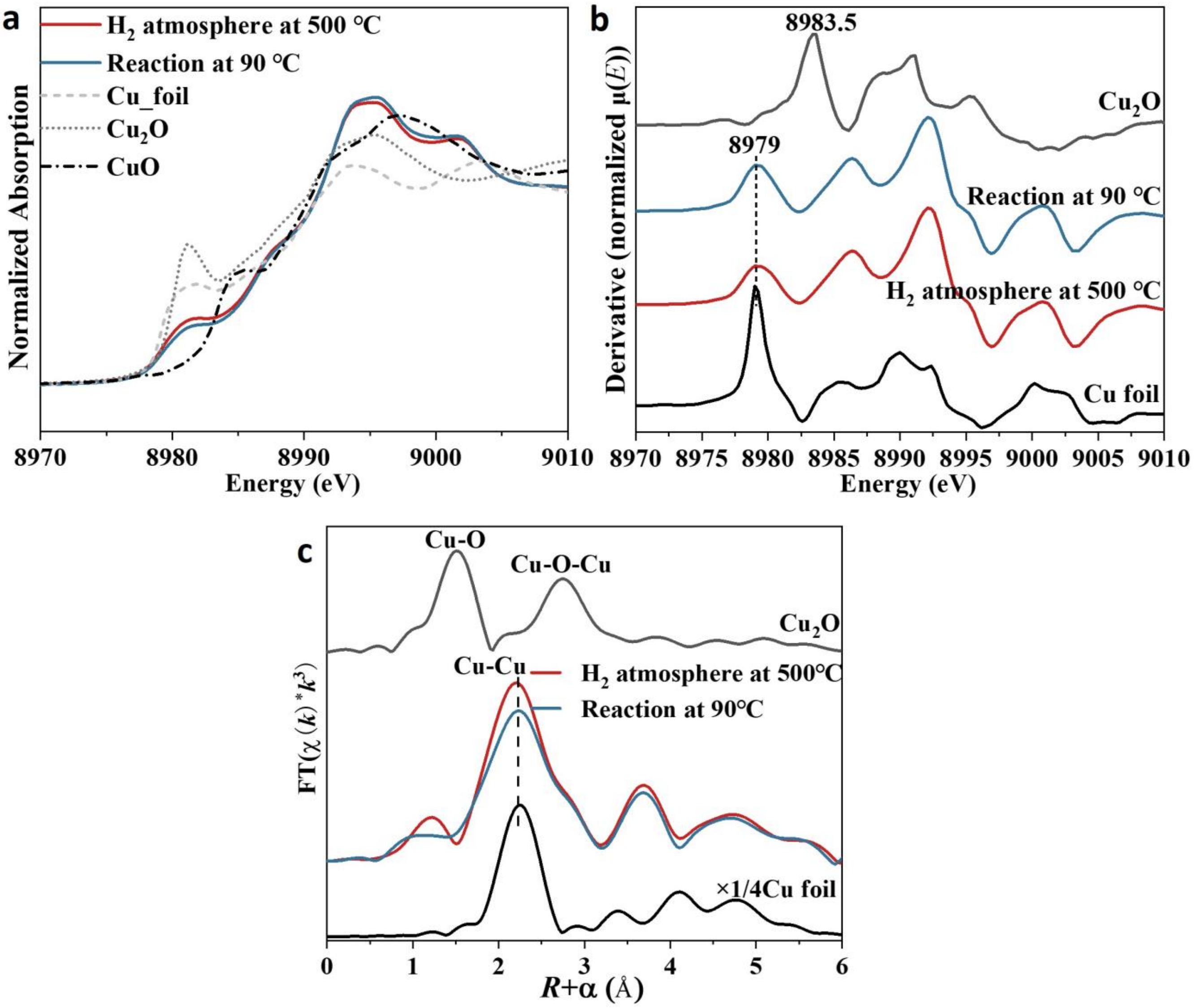

Supplementary Fig. S12. (a) In-situ Cu K-edge XANES profiles, (b) The Cu K-edge Derivative XANES spectra of Cu/MgO catalysts and the references, (c) Cu K-edge EXAFS profiles of Cu/MgO catalysts (The data are k^3-weighted and not phase-corrected).

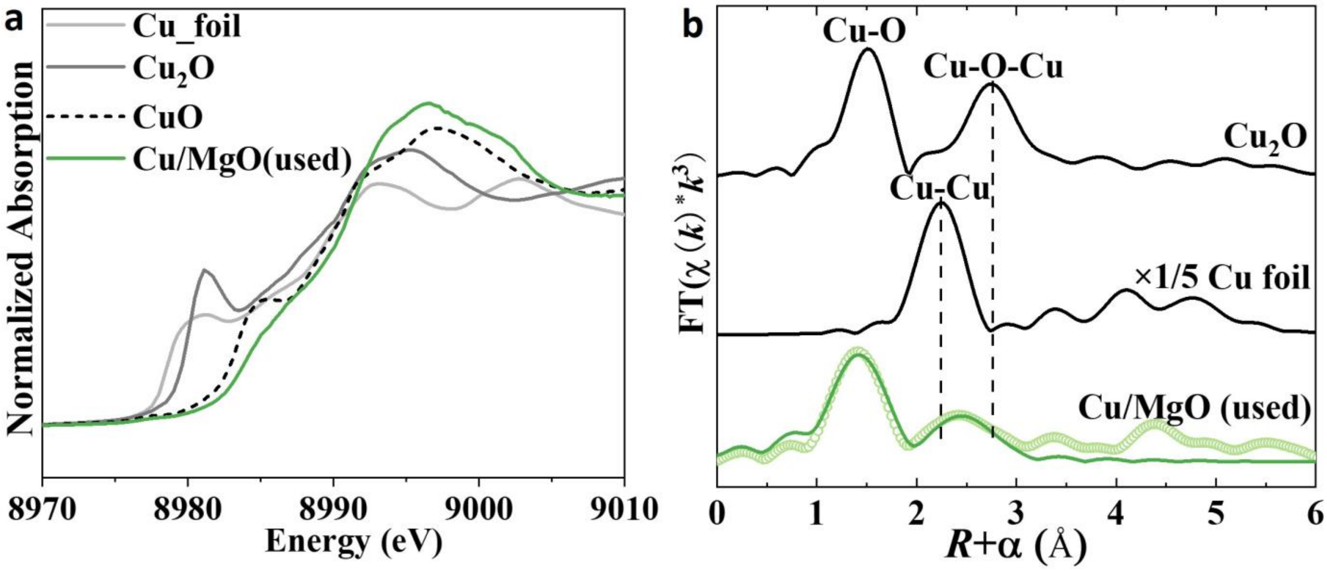

Supplementary Fig. S13. (a) Cu K-edge XANES profiles, (b) Cu K-edge EXAFS profiles of Cu/MgO catalysts (the data are k^3-weighted and not phase-corrected, the curve-fit was generated using the parameters in Supplementary Table S3).

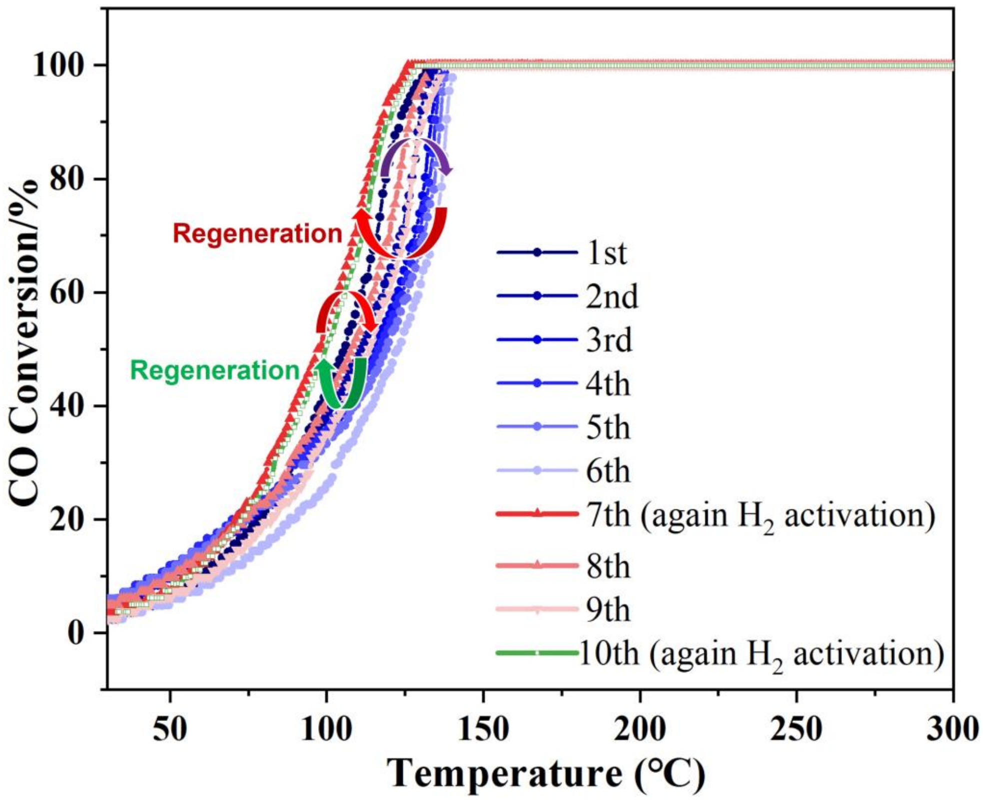

Supplementary Fig. S14. multiple cycles of CO oxidation tests for PtCu/MgO catalysts.

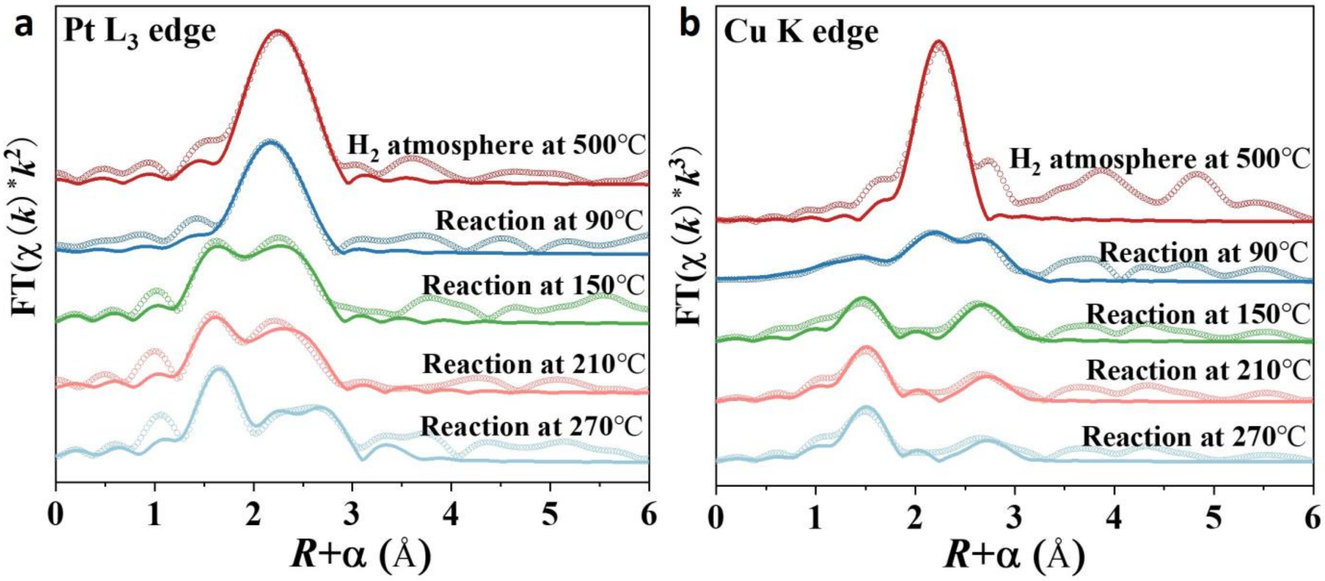

Supplementary Fig. S15. (a) In-situ Pt L_3-edge XAFS (points) and cure-fit (line) of PtCu/MgO (The data are k^2-weighted and not phase-corrected, the curve-fit was generated using the parameters in Supplementary Table S6), (b) In-situ Cu K-edge XAFS (points) and cure-fit (line) of PtCu/MgO (The data are k^3-weighted and not phase-corrected, the curve-fit was generated using the parameters in Supplementary Table S7).

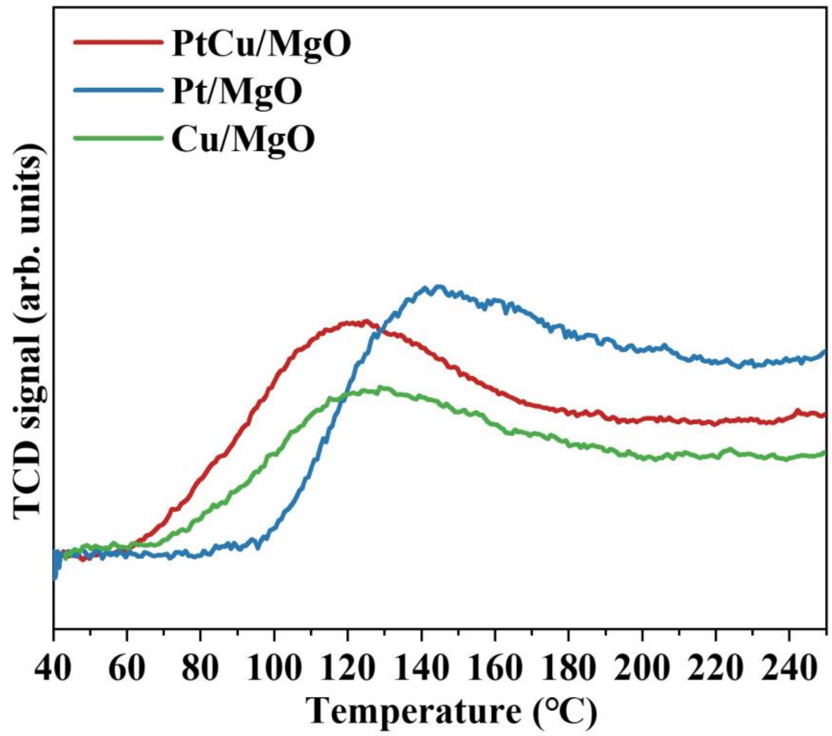

Supplementary Fig. S16. O_2-TPD profile of Pt/MgO, Cu/MgO and PtCu/MgO.

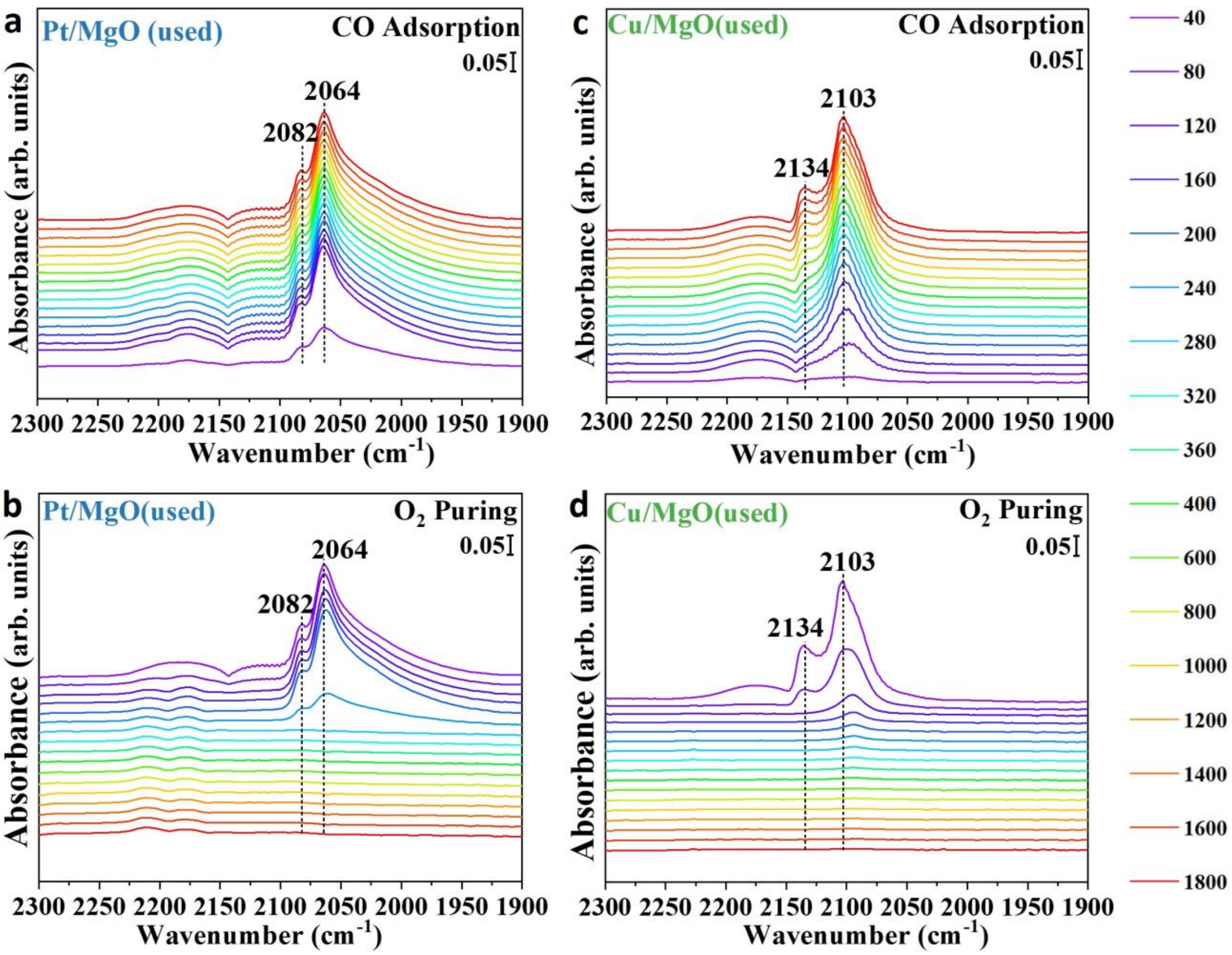

Supplementary Fig. S17. (a-d) In-situ DRIFTS spectra for used Pt/MgO and Cu/MgO catalysts tested at 100 °C: (a-b) Pt/MgO (used), (c-d) Cu/MgO (used). The catalysts were pretreated in situ at 500 °C under H_2 flow and underwent CO oxidation reaction from 30 to 300 °C in the DRIFTS reaction cell before data collection.

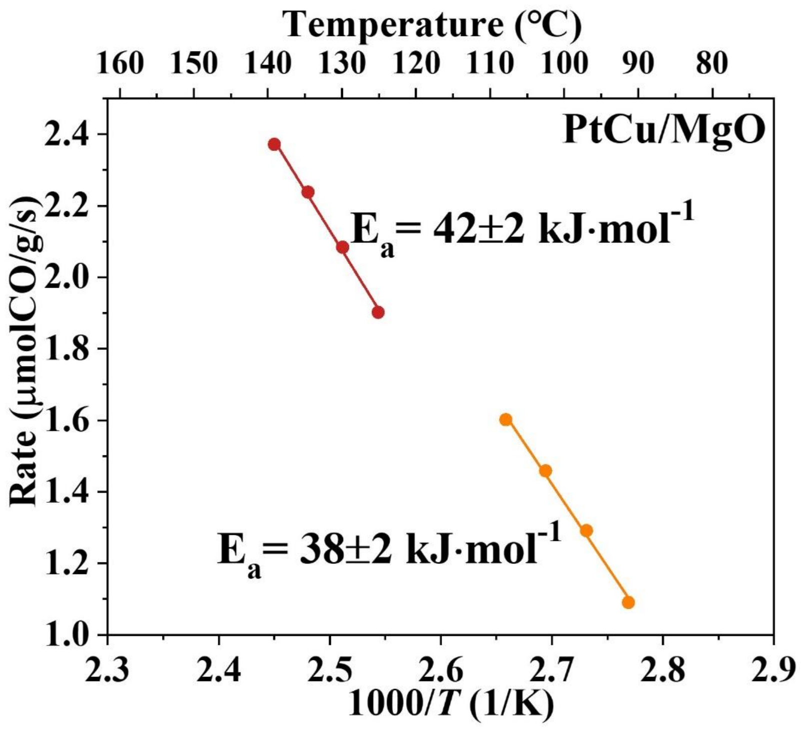

Supplementary Fig. S18. Arrhenius plots for the CO Oxidation over PtCu/MgO at different temperature ranges.

Supplementary Table S1 Pt and Cu bulk concentration, BET Specific surface areas (S_{BET}), BJH pore volume (V_p), lattice constants (a) and average particle sizes calculated from XRD (D_{XRD}) for PtCu/MgO catalysts

Sample	Pt(at.%)[a]	Cu(at.%)[a]	S_{BET} (m^2g^{-1})[b]	V_P (cm^3g^{-1})[b]	a (Å)[c]	D_{XRD} (nm)[c]
MgO	—	—	56	0.49	4.1986(4)	19
Pt/MgO	0.48	—	165	0.83	4.2159(4)<br>4.2185(4)[d]	11<br>11[d]
Cu/MgO	—	6.0	63	0.51	4.2139(4)<br>4.1998 (4)[d]	18<br>18[d]
PtCu/MgO	0.54	5.3	68	0.54	4.2143(4)<br>4.2015(4)[d]	17<br>19[d]

[a] Determined by ICP-AES; [b] Calculated from nitrogen adsorption-desorption results; [c] Calculated from XRD patterns; [d] Used after CO oxidation reaction.

Supplementary Table S2 Averaged oxidation state of platinum (δ) and the corresponding EXAFS fitting results for Pt L_3 edges (R: distance; CN: coordination number; σ^2: Debye-Waller factor; ΔE_0: inner potential correction) of PtCu/MgO catalysts.

Smple	δ^a	Scatter	R(Å)	CN	σ^2	ΔE_0(eV)
PtCu/MgO	3.8	O_1	1.98±0.01	5.5±0.5	0.003	8.4±1.7
		Cu	3.10±0.02	2.8±0.8	0.005	
		O_3	3.66±0.02	10.5±2.9	0.003	
Pt/MgO	4	O_1	2.02±0.01	5.3±0.3	0.003	14.6±0.6
		O_2	3.14±0.01	6.8±0.8	0.003	
		Pt_1	3.37±0.01	5.0±1.4	0.008	
		Pt_2	3.86±0.01	9.3±3.0	0.008	
Pt/MgO(used)	1.0	O	2.02±0.03	1.7±0.4	0.002	11.7±3.7
		Pt	2.77±0.03	6.0±0.9	0.005	
		Pt_1	3.29±0.07	2.6±1.3	0.007	

[a]Determined by linear combination analysis on the XANES profiles with references of Pt foil (δ = 0)/PtO_2 (δ = 4) for Pt L_3 edges. S_0^2 was fixed at 0.88. The σ^2 values were constrained in order to decrease the number of fit parameters and the correlations between them. The distances for Pt-O, Pt-Cu, Pt-Pt are from the crystal structure of PtO_2, $CuPtO_2$ and Pt.

Supplementary Table S3 Averaged oxidation state of platinum (δ) and the corresponding EXAFS fitting results for Cu k edges (R: distance; CN: coordination number; σ^2: Debye-Waller factor; ΔE_0: inner potential correction) of catalysts.

Smple	δ^a	Scatter	R(Å)	CN	σ^2	ΔE_0(eV)
Cu/MgO	2	O	1.97±0.01	2.0±0.2	0.003	-3.8±1.0
		Cu_1	2.84±0.02	0.5±0.3	0.006	
		Mg	2.95±0.03	4.7±0.7	0.008	
PtCu/MgO	2	O	1.96±0.01	2.0±0.2	0.003	-6.1±1.0
		Cu_1	2.81±0.01	0.6±0.3	0.006	
		Mg	2.93±0.02	5.4±0.8	0.008	
Cu/MgO(used)	2	O	1.91±0.01	2.1±0.2	0.003	-7.9±1.3
		Cu	2.55±0.02	0.7±0.2	0.005	
		Cu_1	3.09±0.01	1.5±0.3	0.007	

[a]Determined by derivative of the XANES profiles with references of Cu foil (δ = 0) /CuO (δ = 2) for Cu K-edges. S_0^2 was fixed at 0.93. The σ^2 values were constrained in order to decrease the number of fit parameters and the correlations between them. The distances for Cu-Cu, Cu-O and Cu-O-Mg are from the crystal structure of Cu foil, CuO and Mg_3CuO_4.

Supplementary Table S4 Comparison of the apparent activation energy over the representative Pt-based and Cu-based catalysts for CO oxidation.

Sample	Apparent activation energy ($kJ \cdot mol^{-1}$)	Ref.
1wt%Pt/CeO_2	45	1
0.7wt%Pt/SiO_2	60	2
4wt%Pt/Al_2O_3	70	3
1wt%Pt/SiO_2	98	4
20wt%Cu/TiO_2	64-76	5
10wt%Cu/CeO_2	60-72	6

Supplementary Table S5 Comparison of the activities over the representative catalysts for the oxidation of CO.

Catalysts	Pt(wt.%)	Gas feed composition	Gas hourly space velocity (GHSV)/mL·g_{cat}^{-1}·h^{-1}	$T_{50\%}$	Ref.
Pt/CeO_2	0.5	2vol.% CO/2vol.% O_2/ Ar	150,000	210℃	7
Pt/CeO_2-Al_2O_3	1	1vol.% CO/1vol. % O_2/Ar	200,000	135℃	8
PtCu/Al_2O_3	0.4	1vol.CO/air	12,000	142°C	9
PtCu/TiO_2	1.6	1vol.CO/air	12,000	140°C	9
PtCu/SiO_2	0.3	1vol.CO/air	12,000	143 °C	9
1Pt2Bi/SiO_2	0.9	1vol. %CO/20vol. %O_2/N_2	134,000	85℃	4
PtCu/MgO	0.5	1vol. %CO/20vol. %O_2/He	120,000	100℃	This work

Supplementary Table S6 Averaged oxidation state of platinum (δ) and the corresponding EXAFS fitting results for Pt L_3 edges (R: distance; CN: coordination number; σ^2: Debye-Waller factor; ΔE_0: inner potential correction) of PtCu/MgO catalysts.

Smple	δ^a	Scatter	R(Å)	CN	σ^2	ΔE_0(eV)
PtCu/MgO (H_2 atmosphere at 500℃)	0	Cu	2.61±0.01	11.8±0.8	0.012	5.3±2.2
PtCu/MgO (Reaction at 90℃)	0.5	Cu	2.58±0.01	10.6±0.6	0.014	-0.6±1.9
PtCu/MgO (Reaction at 150℃)	0.8	O	2.04±0.01	1.6±0.6	0.003	1.5±0.7
		Cu	2.64±0.01	7.4±1.6	0.013	
PtCu/MgO (Reaction at 210℃)	0.9	O	2.03±0.03	1.6±0.6	0.003	6.5±2.2
		Cu	2.65±0.01	6.0±1.3	0.013	
PtCu/MgO (Reaction at 270℃)	1.8	O	2.04±0.01	2.1±0.3	0.001	12.8±3.0
		Cu	2.72±0.01	4.6±1.0	0.013	
		Pt	3.18±0.01	2.7±1.7	0.006	

[a]Determined by white line peak height on the XANES profiles with references of Pt foil (δ = 0)/PtO_2 (δ = 4) for Pt L_3 edges. S_0^2 was fixed at 0.87. The σ^2 values were constrained in order to decrease the number of fit parameters and the correlations between them. The distances for Pt-Pt, Pt-O, Pt-Cu are from the crystal structure of Pt foil, PtO_2, and PtCu.

Supplementary Table S7 Averaged oxidation state of platinum (δ) and the corresponding EXAFS fitting results for Cu K edges (R: distance; CN: coordination number; σ^2: Debye-Waller factor; ΔE_0: inner potential correction) of PtCu/MgO catalysts.

Smple	δ^a	Scatter	R(Å)	CN	σ^2	ΔE_0(eV)
PtCu/MgO (H_2 atmosphere at 500℃)	0	Cu	2.59±0.02	6.3±0.9	0.008	3.0
PtCu/MgO (Reaction at 90℃)	0.2	O	1.88±0.04	0.9±0.5	0.003	-9.1
		Cu	2.51±0.02	1.6±0.5	0.007	
		Cu	3.02±0.02	2.3±0.8	0.007	
PtCu/MgO (Reaction at 150℃)	0.4	O	1.90±0.02	1.5±0.5	0.003	-7.6
		Cu	3.05±0.02	2.3±0.8	0.008	
PtCu/MgO (Reaction at 210℃)	2	O	1.93±0.02	1.9±0.4	0.003	-4.9
		Cu	3.09±0.03	1.5±0.7	0.008	
PtCu/MgO (Reaction at 270℃)	2	O	1.92±0.02	1.9±0.4	0.003	-4.8
		Cu	3.09±0.03	1.3±0.7	0.008	

[a]Determined by derivative of the XANES profiles with references of Cu foil (δ = 0) /CuO (δ = 2) for Cu K-edges. S_0^2 was fixed at 0.88. The σ^2 values were constrained in order to decrease the number of fit parameters and the correlations between them. The distances for Cu-Cu and Cu-O are from the crystal structure of Cu foil and Cu_2O.

Supplementary References

1. Zhang, Z. et al. Memory-dictated dynamics of single-atom Pt on CeO_2 for CO oxidation. *Nat. Commun.* 14, (2023).
2. Almana, N. et al. Design of a core-shell Pt-SiO_2 catalyst in a reverse microemulsion system: Distinctive kinetics on CO oxidation at low temperature. *J. Catal.* 340, 368-375, (2016).
3. Lee, S. et al. Manganese Oxide Overlayers Promote CO Oxidation on Pt. *ACS Catal.* 11, 13935-13946, (2021).
4. Nan, B. et al. Unique structure of active platinum-bismuth site for oxidation of carbon monoxide. *Nat Commun*. 12, 3342, (2021).
5. DeSario, P. A. et al. Low-temperature CO oxidation at persistent low-valent Cu nanoparticles on TiO_2 aerogels. *Appl. Catal. B Environ.* 252, 205-213, (2019).
6. Ahasan, M. R., Wang, Y. & Wang, R. In situ DRIFTS and CO-TPD studies of CeO_2 and SiO_2 supported CuO_x catalysts for CO oxidation. *Mol. Catal.* 518, (2022).
7. Yoon, S. *et al.* Influence of the Pt size and CeO_2 morphology at the Pt-CeO_2 interface in CO oxidation. *J. Mater. Chem. A* 9, 26381-26390 (2021).
8. Xie, S. *et al.* Pt Atomic Single-Layer Catalyst Embedded in Defect-Enriched Ceria for Efficient CO Oxidation. *J. Am. Chem. Soc.* 144, 21255-21266 (2022).
9. Qin, H., Qian, X., Meng, T., Lin, Y. & Ma, Z. Pt/MO_x/SiO_2, Pt/MO_x/TiO_2, and Pt/MO_x/Al_2O_3 Catalysts for CO Oxidation. *Catal.* 5, 606-633 (2015).
